# Socio-ecological model as a framework to understand the low participation of Earth Hour among Chinese college students: conflict between belief and practice

**DOI:** 10.3389/fpsyg.2024.1288711

**Published:** 2024-05-06

**Authors:** Keqin Yin, Yihui Wang, Huixin Xu, Man Lei

**Affiliations:** ^1^Business College, Shanghai Normal University, Shanghai, China; ^2^Information, Mechanical and Electrical Engineering College, Shanghai Normal University, Shanghai, China; ^3^Foreign Languages College, Shanghai Normal University, Shanghai, China

**Keywords:** socio-ecological model, Earth Hour, participation, college students, pro-environmental behavior

## Abstract

Earth Hour, a global mass effort coordinated to show concern for green urban construction and sustainable development, was first organized by the World Wildlife Fund in Australia in 2007 with a growing trend of participation worldwide. However, analysis of participation in Earth Hour based on a large population are sparse, with only a few studies reporting details in positive results without a clear pattern that explains the potential low participation. This study focuses on the non-participants and analyzed the reasons for low participation in Earth Hour using a questionnaire with 401 college students based on the socio-ecological model. Two aspects are explored: (1) social-demographic features; (2) psychosocial traits (environmental awareness, acceptance for law, social support from family and friends and knowledge about the event). Barriers toward participation are included as mediators to explain how these basic features change students’ decision on joining large-scale environmental campaign. A participation analysis method using binary logistic regression and one-way MANOVA is applied in data analysis. This study highlights that the irrelevance between students’ belief and practice on environmental protection should not be overlooked, and that college students are inclined to join in groups in relevant activities—conversely, herd effect could greatly reduce their willingness to participation. The findings of this study have wider implications for school educators, practitioners and organizations involved in pro-environmental career. This paper highlights that, from an international perspective, the essence of collective action with a similar nature to Earth Hour and contributes to a global dialogue on fostering sustainable behaviors.

## 1 Introduction

Organized by the World Wildlife Fund and partners as a symbolic lights-out event in Sydney in 2007, Earth Hour is now one of the largest grassroots movements for the environment (Chan et al., 2020). Held every year on the last Saturday of March, Earth Hour engages millions of people in more than 180 countries and territories, calling for switching off non-essential electric lights for a single hour of 1 day every year to show support for our planet under climate change (Jechow, 2019). Apart from its significant contribution to raise public awareness of environmental issues, Earth Hour has reduced electricity consumption an average of 4% from 2008 to 2014, leading a fashion of low carbon lifestyle worldwide (Olexsak and Meier, 2014).

Earth Hour was first introduced to China in 2009, with Baoding being the first city to participate officially (Feng, 2009). By 2013, this annual lights-out event took place in 127 cities, including landmarks of more than 4 first-tier cities (e.g., Bird-nest in Beijing, Oriental Pearl TV Tower in Shanghai), eastern China (Environmental Protection Publicity and EDU Center of Canton, 2013). Nonetheless, it remains doubtful whether this event had been put into practice among Chinese citizens, or simply limited to a false image built by local institutions. An investigation by HuiCong D&B Market Research company spoke highly of the positive feedback from the public in Earth Hour (an estimate of 67.3% individual participation rate with a total sample of 4,408 in PRC) (2011), which was believed to be the result of the effective top-down measures. However, when it comes to individual participation, a totally converse picture was shown in Wang’s study (2012). Nearly 90% of the respondents heard of Earth Hour, but 2 out of 3 are non-participants among a smaller sample featuring the participation in suburban area (Wang, 2015).

Certain research has reached an agreement on a relatively low participation rate of Earth Hour in China in recent years, indicating a descending trend of individual participation with the change of time. Compared with the positive feedback in 2011, an average participation rate of 2.7 times (out of 8 times) by 2015 was found in Chinese citizens, with 24.2% of them never participated in this event; citizens aged above 55 or with a higher education level outweighed their counterparts in long-term participation (Wang, 2015). Among non-participants, the effect of reducing electricity consumption in Earth Hour was controversial (Solomon, 2008; Vuong et al., 2020), not to mention the insufficient generation requirements and potential grid failure triggered by sharp drops and peaks of electricity use (Olexsak and Meier, 2014). Given the ambiguous findings in both individual participation and citizens’ three-minute passion for this activity, it remains essential to carry out this classic research 15 years later since the first launch of Earth Hour.

One of the difficulties in conducting this research is that there is no valid standardized set of reasons accounting for low participation associated with environmental protection nationwide and most results end up in the form of details, adding up obstacles to discover a regular pattern. Given the limited evidence available, we could not predict the respondents’ feedback but applied the bottom-up approach. To ensure the validity of the study, social-ecological participation analyzing model (Van Dyck et al., 2017) were used as reference in methodology, with a more accurate sample aiming at college students only.

However, existing academic works are not yet sufficient to explain the potential mechanism leading to the non-participation in Earth Hour, especially the case unique to PRC. Relevant studies mostly focus on small samples and case analysis, ignoring the fact that they are looking into a large-population-based event. By including more respondents in the sample, this study has adopted more quantitative methods which are known to be common in empirical research. Also, it is the first time that socio-ecological model is used in analyzing the participation of pro-environmental events in Asian context, where local regulations and the interplay between different social groups are concerned. Distinguished from other general reports, this paper focus on the respondents who are more likely to underperform based on their extreme scores on certain social psychological features, to figure out more specific reasons indicating low participation rate.

The present study addresses four research questions:

(1) whether there are more non-participants than participants among Chinese college students in Earth Hour?
(2) which socio-ecological factors are related to participation in earth hour event?
(3) how do barriers vary between different subgroups of gender, family income, living environment and educational level?
(4) what are the most prevalent barriers toward participation in the total sample and in students with psychosocial characteristics associated with extreme odds?

## 2 Literature review

### 2.1 Conflict between belief and practice

The conflict between belief and practice does exist and has been extensively explored in literature (Desforges and Cockburn, 1987; Farrell and Lim, 2005; Yang, 2019; Lei and Medwell, 2021). Belief was referred to as messy constructs by Pajares (1992), inconsistent with the observed practices in studies concerning teaching behaviors (Duffy and Anderson, 1984).

Likewise, a problem in large-scale environmental protection campaigns unique to China could be the imbalance between its good will and low efficiency to carry out (Liu and Diamond, 2008; Jin et al., 2023). Particularly, the lack of a meaningful institutional framework to allow public participation deserves a bit more reflection in environmental protection (Li et al., 2012). For one thing, the publicity of Earth Hour has been ramping up nationwide, with 127 cities joining this campaign in 2013 which quadrupled the number three years ago (Gu, 2017). With a rapid surge of bus advertisements and celebrity endorsement in the past decade, MCI (i.e., Media Communication Index) for Earth Hour has hit a new record high of 79.9% via printed and online media (HuiCong D&B Research, 2011; Gu, 2017). For another, a few critics have pointed out that some of the college students might place their passion for environmental protection on the “wrong” side, resorting to enterprises and media for funds and fame before they ensure the feasibility and social benefits of their projects (Zhang, 2001; Jin et al., 2023). Despite that the existing research set a solid theoretical foundation for our study, few studies investigate the conflict between belief and practice among college students nationwide in this matter.

### 2.2 Socio-ecological model

#### 2.2.1 Definition and development

The Socio-ecological Model (SEM) was a concept first suggested by Bronfenbrenner (1977) as an ecological systems theory for human development and was later redefined by McLeroy et al. (1988) as a framework to promote health-related behavioral change. Socio-ecological models were introduced to urban studies by sociologists associated with the Chicago School after the First World War as a reaction to the narrow scope of most research conducted by developmental psychologists. These models bridge the gap between behavioral theories that focus on small settings and anthropological theories.

**Figure 1:**
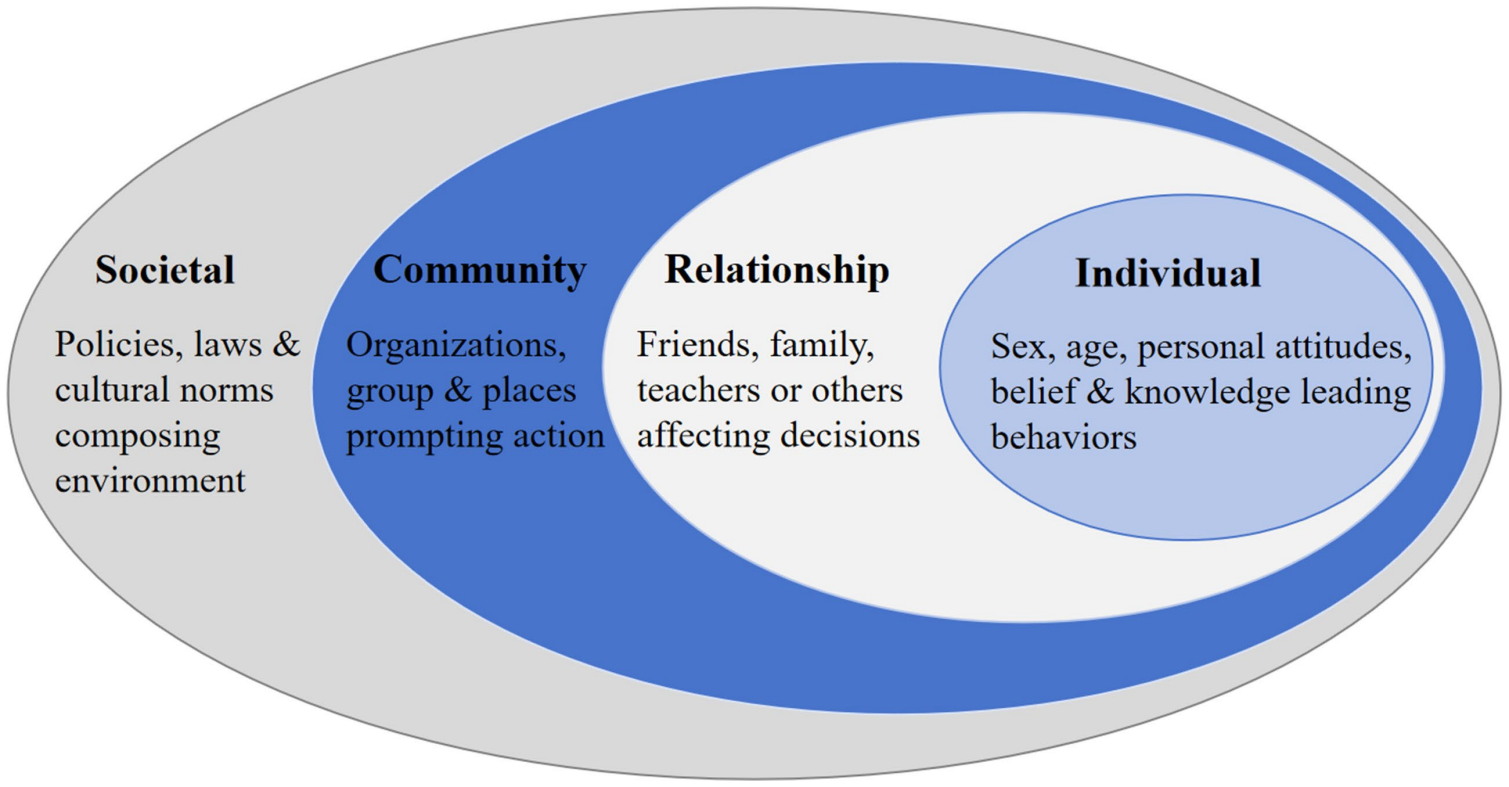
The socio-ecological model for general purposes.

The initial theory by Bronfenbrenner was illustrated by nesting circles that place the individual (sex, age, etc.) in the center surrounded by various systems: microsystem (family, peers, school, church), mesosystem (interplay inside of microsystem), exosystem (industry, mass media, social services, neighbors, local politics) and macrosystem (attitudes and ideologies of the culture). The SEM stated that health is affected by the interaction between the characteristics of the individual, the community, and the environment that includes the physical, social, and political components (Kilanowski, 2017). The model was developed to further the understanding of the dynamic interrelations among various personal and environmental factors, and their impact on a specific type of individual’s behavior. Generally, it contained four dimensions: Individual, Interpersonal/Relationship, Organizational/Community and Societal (see Figure 1).

#### 2.2.2 Applications of SEM in pro-environmental studies

Previous studies have proved that SEM can be applied to a variety of pro-environmental engagement analysis. Environmentally sustainable behavior (ESB) was interpreted as involving the way that we interact with environmentally relevant things (e.g., automobiles, trashcans, lawn sprinklers, lights, and home heating systems) around us in our everyday lives (Hormuth, 1999). Kurz suggested a social-ecological framework for promoting ESB (2002) to understand and change ESB. It combines Hormuth (1999) ecopsychological approach to ESB and Baron and Misovich (1993) social-ecological framework of attitude and behavioral change. A study of pro-environmental behavior (PEB) among wildscape gardeners (Jones et al., 2021) has shown that applied factors from SEM concerning individual, social environment, physical environment, and policy level would provide a robust result in logistic regression. Smith et al. (2021) research explained how individuals’ attachment to community affect their perceived social norms and thus, changing their perception of climate change risk and subsequent willingness to engage in pro-environmental behavior, in a context which is closer to Earth Hour.

According to McLeroy et al. (1988), SEM assumes that appropriate changes in the social environment will produce changes in individuals, and that the support of individuals in the population is essential for implementing environmental changes. Earth Hour operates on the premise that by orchestrating a globally synchronized hour of reduced energy consumption, it can induce a shift in social norms regarding energy usage. Furthermore, the success of Earth Hour relies heavily on the active participation and support of individuals worldwide. Therefore, Earth Hour aligns with the foundational assumptions of SEM, making it a fitting subject for research within the SEM framework.

#### 2.2.3 Socio-ecological factors unique to earth hour

Socio-ecological models of health behavior posit that socio-demographic, psychological, social, and environmental characteristics are all important determinants of health behaviors (Sallis et al., 2015), and can impact participation in pro-environmental events as well (Kurz, 2002).

Some evidence is available regarding the socio-demographic profile of participants, compared with non-participants. Female presented as a more pro-environmental gender in China (Li et al., 2022), since females were more concerned with environmental problems and more supportive of plastic-ban policies; however, the gender gap of PEB is not so apparent among university students (Vicente-Molina et al., 2018). Specifically, in PRC, living environment played a significant role in Earth Hour public engagement. Wang (2015) mentioned that fewer participants were found in rural area where publicity of Earth Hour was rather low; while lately more active and frequent participants were found in third-tier and fourth-tier cities with an extra 13% growth rate compared with metropolitans (Zhuang, 2023). Zhou and Fan (2020) stated that the average income was positively associated with educational level throughout the past two decades in PRC, however, the participation rate for low-income individuals have been increasing in recent years.

Psychological aspects like perceived barriers and motivation, and social aspects can be framed within the Identity Theory (Stryker and Burke, 2000) and Theory of Planned Behavior (TPB) (Ajzen, 1985). Studies have shown that individuals who have a stronger environmental self-identity express stronger pro-environmental intention and perform pro-environmental actions more frequently (Barbarossa et al., 2015; Yang and Li, 2021), thus environmental awareness was introduced to our model. Chan et al. (2020) was established on TPB and suggested that Earth Hour participation is determined by intention, that is in its turn determined by attitude (individual’s favorable/unfavorable evaluation of the behavior), behavioral control (perceived ease or barriers to perform the behavior) and subjective norms (perceived pressure from important others). In specific, “social support from family and friends” was measured as subjective norms, due to its significant boost on pro-environmental behaviors in Yang and Li (2021) and college students’ engagement in group events (Limniou et al., 2022). “Similarities with other events” was included as behavioral control, given that participants tend to become less motivated and underestimate the importance of an event when it shares a high proximity with other events in PEB studies (Margetts and Kashima, 2017; Chatelain et al., 2018). “Disappointment/doubts in effectiveness” was quired as attitude since existing studies (Solomon, 2008; Vuong et al., 2020) mentioned the controversial effect of energy saving in Earth Hour, and it is believed that such controversy could reduce people’s passion to engage.

## 3 Methodology

### 3.1 Procedure and participants

This study involved administrating a questionnaire to 401 college students in the PRC. The study was approved to be within the Code of Ethics followed by the collaborators’ universities. College students in the PRC were asked to complete an online questionnaire, 372 of the 401 questionnaires distributed were returned (a very high return rate of 93%). The questionnaire had four parts. The first part aimed to sort students by their frequency of participation within 5 years and separate non-participants from the total sample. The second part and third part aimed to capture respondents’ demographics and psychosocial features, respectively. The fourth part of the questionnaire examined the significance of each barrier toward participation perceived by respondents. The systematic design and analysis were based on a study concerning various factors as correlates of non-participation in running events (Van Dyck et al., 2017).

The online questionnaires were distributed through an online platform (Wenjuanxing website) to college students within 12 prefecture-level cities across the PRC. The items and format were pilot tested with 43 college students (not involved in the main study) and revised based on their comments and suggestions. A cluster sampling technique was used to select the respondents whose educational backgrounds range from first-class universities to vocational schools, and to include a representative proportion of rural, suburban, and urban schools nationwide. Additional respondents were recruited through snowball sampling. The researchers shared the questionnaire links with currently enrolled research participants and encouraged them to spread the project on social media platforms such as WeChat, QQ, and Weibo to capture a growing chain of participants.

The online questionnaire was available from the end of April to mid-May in 2022 and it took 139 s to complete on average. All personal information in the questionnaires were collected anonymously.

### 3.2 Modifications to the model

The study was inspired by Van Dyck et al. (2017) who applied the social-ecological model to non-participation analysis of running events in Belgium. In this paper, several modifications were made regarding that the features of sample are unique to Chinese college students and environment-related activities.

For socio-demographic variables, age was excluded from the initial model since a four-year-range among undergraduates was short enough to be neglected in this matter. Monthly family income was added to demonstrate how students’ main economic sources affected their choice in participation. Educational level was further divided into undergraduate and junior college for college students. For psycho-social variables, social support from family and from friends were merged into one factor (“social support from family and friends”), with factors related to personal character introduced (Larson and Lach, 2008). Factors unique to running events were not considered, to name a few, bad physical condition, annoyance spectators and insufficient challenging among barriers toward participation; and min/week MVPA among activity-related variables.

It is worth mentioning that psychosocial factors were assessed in separate questionnaires in the previous research, these questionnaires were adapted to one question using five-point Likert scale, respectively, for each factor in this study.

### 3.3 Measures

#### 3.3.1 An overview of the socio-ecological model

**Figure 2:**
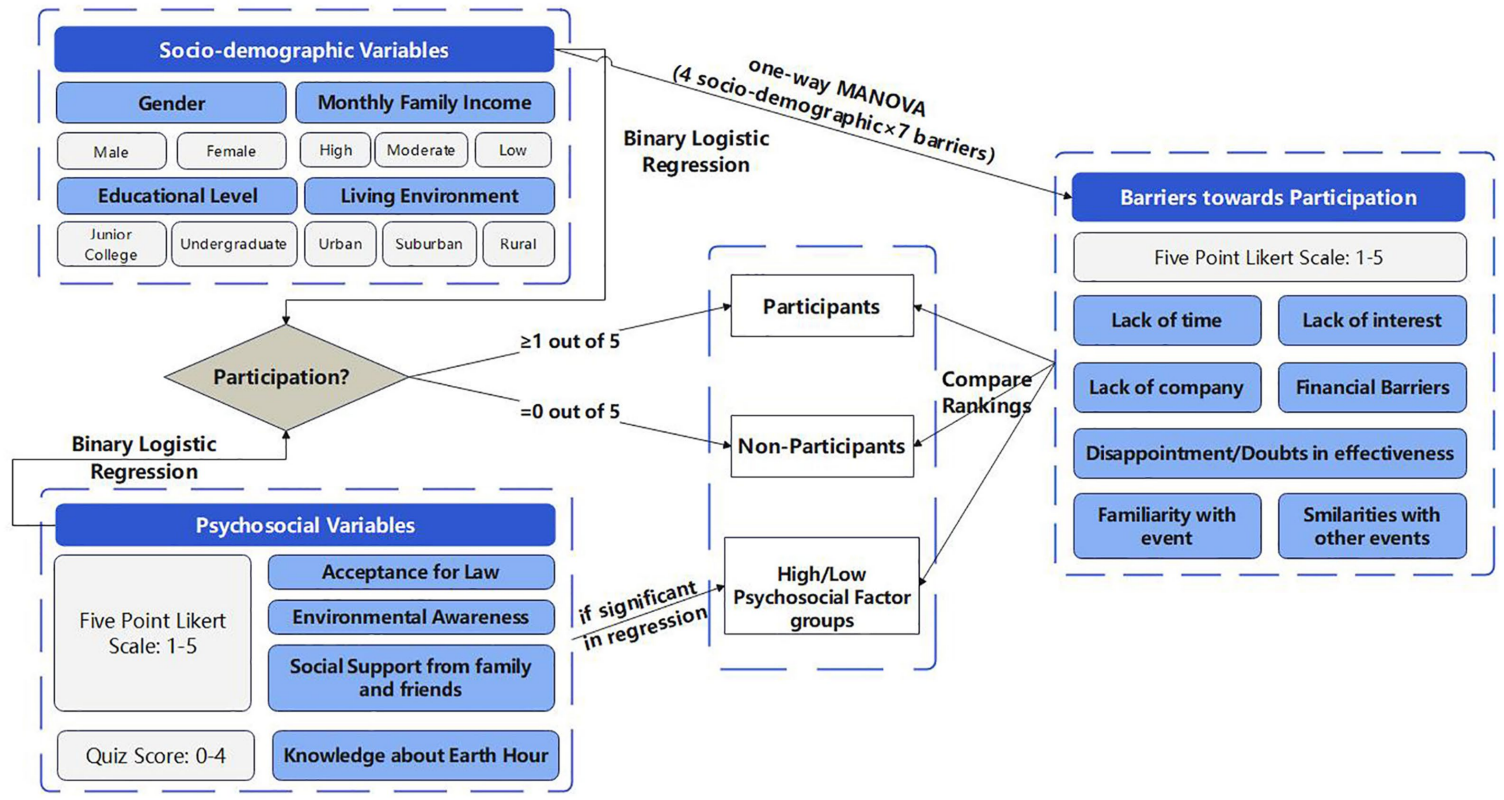
Overview of the present research.

The basic idea of this research is shown as follows (see Figure 2).

#### 3.3.2 Socio-demographic characteristics

The following socio-demographic characteristics were assessed: gender, monthly family income (low, moderate, high, Zhou and Fan, 2020), educational level and living environment.

#### 3.3.3 Psycho-social factors and attitude variables

Four categories of subjective factors were included in the questionnaire: environmental awareness, acceptance for law and regulations, social support from family and friends and knowledge about the Earth Hour event. While the first two factors were concerned with attitude variables depending on personal character (Larson and Lach, 2008), the last two factors were psychosocial which showed how personal choice was influenced by social environment. Detailed standards for selecting these variables have been discussed above (see section 2.2.3).

All factors except knowledge about the Earth Hour event were assessed on a five-point Likert scale from strongly disagree to strongly agree (environmental awareness, acceptance for law and regulations) and from never to very often (social support from family and friends).

Knowledge about the Earth Hour event was assessed by presenting the students an earth hour quiz (CTVNews.ca Staff, 2015) which contained four questions, including the specific time, originated city, organization, and geographic reasons for earth hour. Scores for “knowledge” could range between 0 and 4.

#### 3.3.4 Participation in the Earth Hour event

Participation in the Earth Hour event was assessed by one question: “How often do you participate in the Earth Hour event in the past 5 years?” Due to the convenience of switching indoor lights for an hour, the quality and thoroughness was not considered. Those who gave an answer would be sorted into three levels, non-participants (never participated), occasional participants (participated for 1–2 times) and regular participants (participated for 3–5 times). For the analysis this variable was dichotomized into non-participation (never participated in 5 year) versus participation (at least one time in 5 years).

#### 3.3.5 Barriers toward participation

Participants were asked about potential barriers preventing participation, except for those individuals that did participate for more than two times in this event in the past 5 years. A list of 10 potential barriers was compiled during an expert meeting with two behavioral research scientists and two psychologists and was based on previous research on perceived barriers toward physical activity (De Bourdeaudhuij and Sallis, 2002; Deforche et al., 2004). With minor modifications, these barriers were catered to better fulfill the topic of an environmental protection activity in this paper (see sections 2.2.3 and 3.2).

The following 7 barriers were queried: lack of interest, lack of time, financial barriers, lack of company/encouragement, disappointment and doubts in the effectiveness, low familiarity with the event, too many similarities compared to other environmental protection events. All items were assessed on a five-point Likert scale ranging from impossible to very likely.

### 3.4 Data analysis

Before analysis, all variables with scale data were under preprocessing. Item that contains a Cronbach’s α above 0.7 would be accepted. The results in Table 1 had made it clear that the following items had passed the test and were to be analyzed.

**Table 1:** Reliability analysis on scale data.

No.	Variable	Correlation between the deleted item and the total	Cronbach’s α	Reliability
1	Environmental awareness	0.562	0.856	High
2	Acceptance for law and regulations	0.54	0.835	High
3	Social support from family and friends	0.573	0.836	High
4	Lack of interest	0.534	0.836	High
5	Lack of time	0.576	0.832	High
6	Lack of company	0.746	0.813	High
7	Disappointment/doubts in effectiveness	0.516	0.838	High
8	Familiarity with event	0.659	0.823	High
9	Similarities with other events	0.681	0.82	High

The analysis of the reliability and validity of the data was completed using the Statistical Package of Social Science (SPSS 28.0) and all figures presented through excel. To examine the socio-demographic and psychosocial correlates of participation in Earth Hour, a binary logistic regression analysis was conducted. Participation in the Earth Hour event during the past 5 years (yes/no) was included in the model as the dependent variable; four socio-demographic factors (i.e., gender, monthly family income, educational level, living environment) and four psychosocial variables (i.e., knowledge, social support from family and friends, environmental awareness, acceptance for regulations) were included as independent variables. Descriptive statistics were used to describe the barriers toward participation present in the overall sample and in those students with characteristics related to lower odds of participation in Earth Hour. To examine the differences in barriers toward participation depending on gender (men versus women), monthly family income (low, moderate, high), living environment (urban, suburban, rural) and educational level (undergraduate, junior college), four one-way MANOVA analyses were conducted. Statistical significance was set at *p* < 0.05 for all analyses.

## 4 Results

### 4.1 Descriptive characteristics of the sample

#### 4.1.1 Socio-demographic and psychosocial features in sample

In total, 401 students completed the questionnaire, of which 372 responded effectively to all the questions and were included in the sample. The socio-demographic and psychosocial characteristics of the total sample are listed in Table 2. Among the sample, 174 (46.77%) never participated in this event and 198 (53.23%) participated in Earth Hour for at least one time.

**Table 2:** Descriptive characteristics of the study sample.

Variable	Total sample	Non-participants	Participants
	(*n* = 372)	(*n* = 174)	(*n* = 198)
Socio-demographic variables			
Gender (%)			
Men	30.91	32.76	29.29
Women	69.09	67.24	70.71
Educational level (%)			
Undergraduate	60.48	62.64	58.59
Junior college	39.52	37.36	41.41
Living environment (%)			
Urban	23.12	21.26	24.75
Suburban	26.61	29.89	23.74
Rural	50.27	48.85	51.52
Monthly family income^1^ (%)			
Low income	51.34	53.45	49.49
Moderate income	36.02	35.63	36.36
High income	12.63	10.92	14.14
Psychosocial variables [mean (SD)]			
Environmental awareness^2^	4.54(0.83)	4.56(0.88)	4.53(0.97)
Acceptance for law and regulations^2^	3.72(0.94)	3.59(0.99)	3.83(0.88)
Social support from family and friends^3^	3.02(1.31)	2.56(1.39)	3.42(1.08)
Knowledge about the Earth Hour event^4^	0.78(0.97)	0.80(0.94)	0.77(1.01)

SD = standard deviation. 1: measured by CNY, Low∈[0,5000], moderate∈(5000,10000], high∈(10000,+∞); 2: five-point Likert scale from strongly disagree to strongly agree; 3: five-point Likert scale from never to very often; 4: minimum 0, maximum 4.

Overall, 69.09% of the sample was female, 60.48% had a bachelor’s degree, 23.12% lived in urban area and 50.27% shared a rural dwelling, nearly a half of the respondents were from low-income families.

The overall environmental awareness was relatively high, with a per capita score of over 4 among the total sample, participant, and non-participant groups. However, the overall level of knowledge about Earth Hour was low, with an average score of less than 1, indicating that most students had not answered almost one of the four questions correctly and had insufficient knowledge about this activity.

#### 4.1.2 Interval estimation of low-frequency participation ratio

Participants who participate less than 3 times within 5 years are defined as low-frequency participants. Since the low-frequency participation ratio is a dichotomy variable, there is a normal approximation in the case of a large sample, corresponding to a two-point distribution. Therefore, the confidence interval for the overall ratio at the 95% confidence level can be obtained:

$$p\pm Z_{0.025}\sqrt{\frac{p\left(1-p\right)}{n}}$$

**Figure 3:**
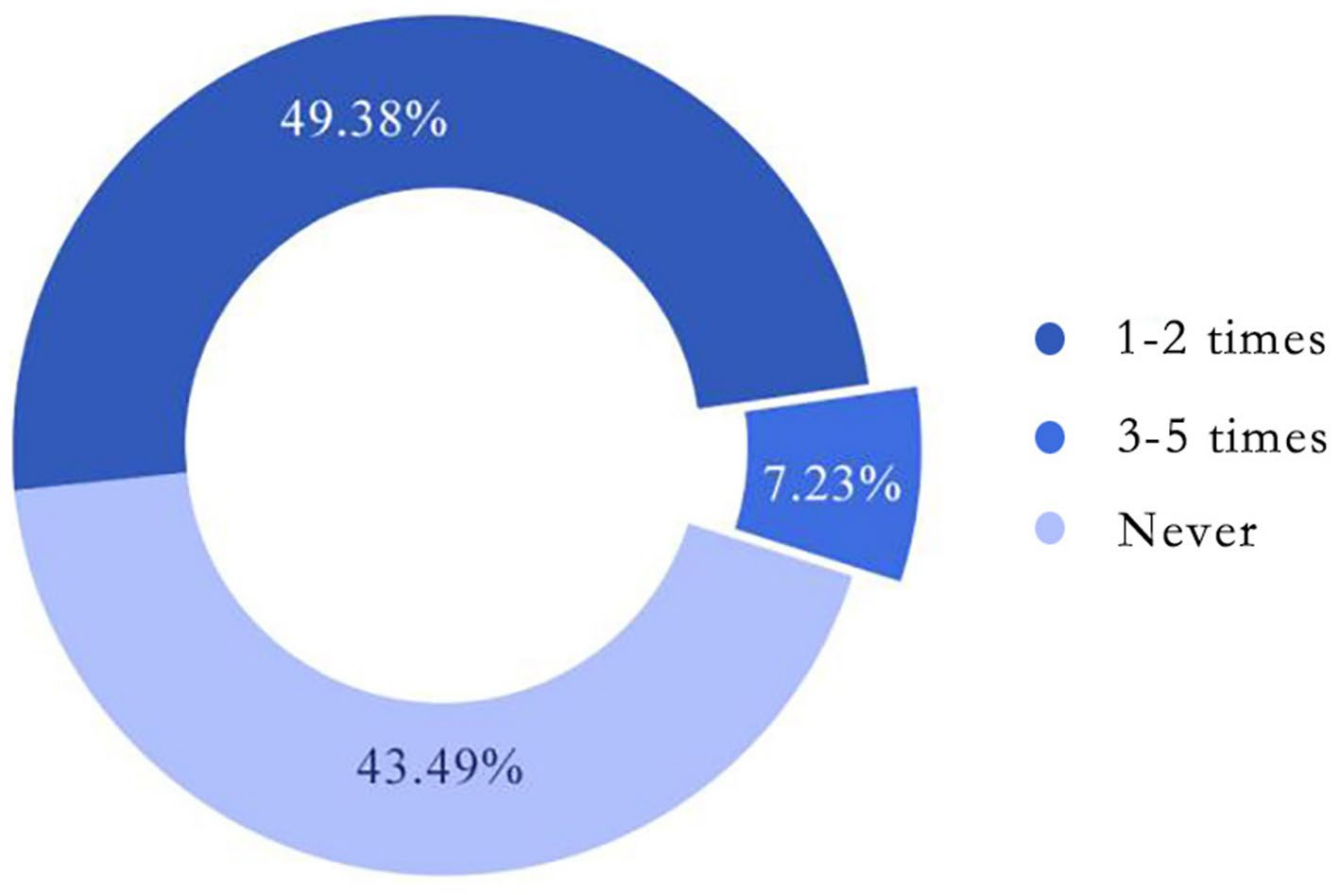
Participation of the original sample (*n* = 401).

In total, 401 students completed the questionnaire, among which the non-participants and occasional participants of the Earth Hour event take up 92.77% (i.e., *p* = 92.77%, *n* = 401) (see Figure 3). As of 2022, the 95% confidence interval of the proportion of non-participants among all Chinese college students in the past 5 years is therefore (38.54, 48.24%), which is above 24.2% non-participation found in previous research by 2015. In this way, it’s safe to draw the conclusion that college students in China share a low participation in Earth Hour in the past 5 years.

### 4.2 Socio-demographic and psychosocial correlates of participation in Earth Hour event

#### 4.2.1 Spearman coefficients between socio-ecological factors

**Figure 4:**
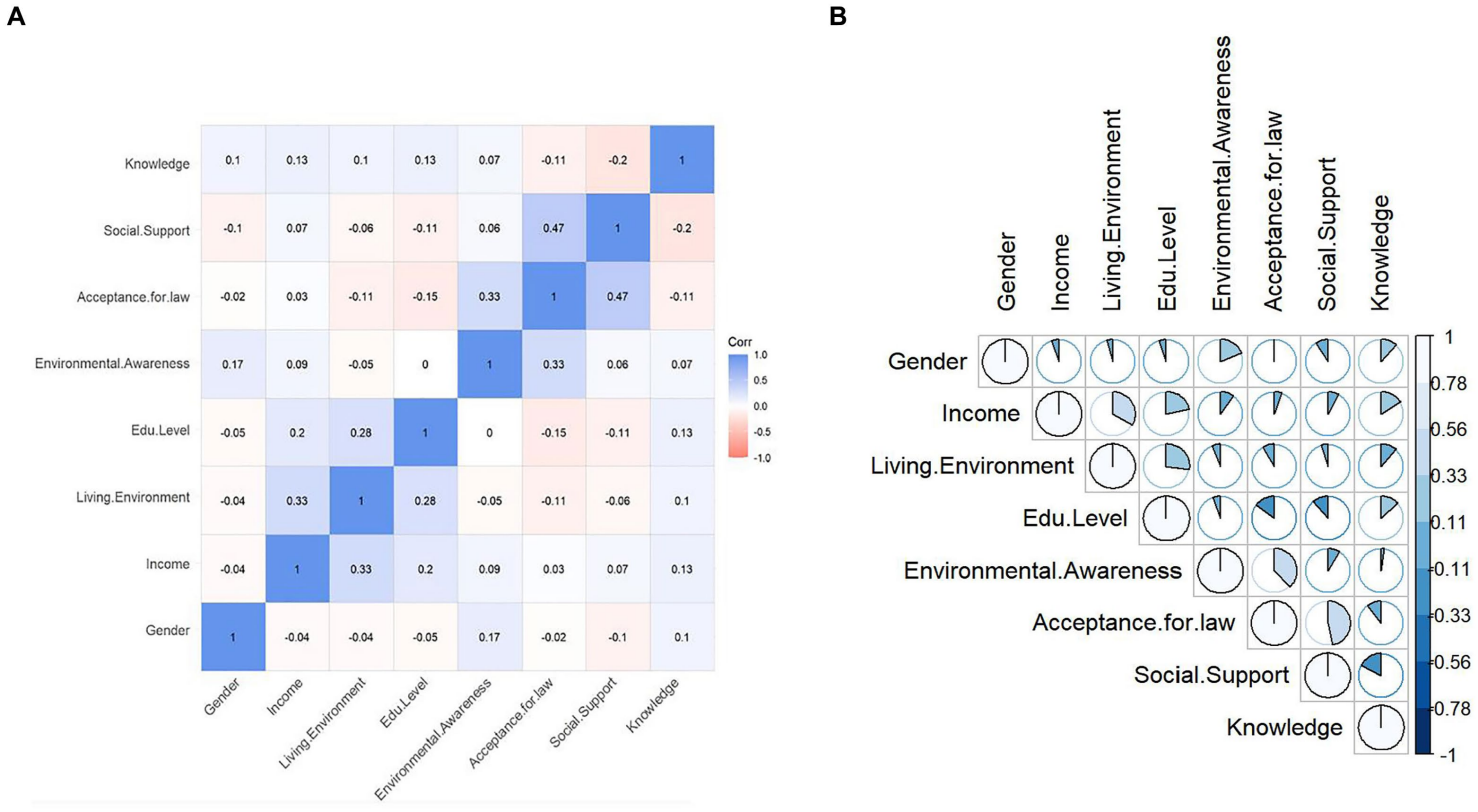
Correlation matrix of socio-ecological variables. **(A)** Hierarchical clustering method. **(B)** Pie chart by Spearman coefficients.

Spearman coefficients were applied for rank correlation between discrete variables (i.e., all socio-demographic and psychosocial characteristics) in our research (see Figure 4). Odds for multicollinearity are very low since no coefficient is larger than 0.5 among significant correlation, which makes it possible for logistic regression.

Acceptance for law is positively related to social support (*ρ* = 0.47, *p* < 0.01) and environmental awareness (*ρ* = 0.33, *p* < 0.01). However, a higher level of knowledge is linked with a lower social support (*ρ* = −0.2, *p* < 0.01); and better-educated students were found to be less tolerant with relevant law (*ρ* = −0.15, *p* < 0.01). A more urbanized dwelling is relevant to higher income and educational level, which is in accordance with common sense.

#### 4.2.2 Binary logistic regression

Since the number of the sample is 10–15 times larger than that of the independent variables (i.e., 372 > 15*8), the binary logistic regression was applied.

Results of the binary logistic regression analysis are shown in Table 3. The analysis revealed that social support from family and friends was significantly associated with participation in Earth Hour event. Students perceiving more social support from family and friends (OR = 1.790, 95% CI = 1.462, 2.192) were more likely to have participated in Earth Hour for at least one time during the past 5 years than their counterparts. For the socio-demographic and the other psycho-social factors, no significant results were found.

**Table 3:** Binary logistic regression analysis of socio-demographic and psycho-social correlates of participation in Earth Hour event.

Dependent variable: participation in Earth Hour: 0 = no participation during the past 5 years, 1 = participation for at least one time during the past 5 years						
Correlate	β	SE	*P*-value	Odds ratio	95%CI	
Environmental awareness	−0.164	0.151	0.279	0.849	0.631	1.142
Acceptance for law	−0.033	0.146	0.821	0.967	0.726	1.289
Social support	0.582	0.103	0.000	1.790	1.462	2.192
Knowledge about event^1^	0.068	0.119	0.566	1.071	0.848	1.351
Gender (ref: male)	−0.383	0.250	0.127	0.682	0.417	1.115
Monthly family income low	−0.259	0.387	0.503	0.771	0.361	1.648
Monthly family income moderate	0.184	0.382	0.631	0.832	0.393	1.761
Living environment suburban	−0.229	0.280	0.413	0.795	0.460	1.376
Living environment urban	0.083	0.302	0.782	1.087	0.602	1.964
Educational level undergraduate	−0.069	0.241	0.776	0.934	0.582	1.498
Constant	−0.468	0.930	0.615	0.626	–	–

SE, standard error; 95%CI, 95% confidence interval. ^1^Range from 1 to 5 with a linear variation.

With a Hosmer significant level below 0.6, however, it remains doubtful whether these correlates were well-explained by the fitting results. Thereby, a further test was taken to investigate the correlation between participation and social support, which suggested a significant result (Pearson Chi-Square = 61.940, *p* < 0.01).

### 4.3 Differences in barriers toward participation depending on gender, family income, living environment, and educational level

Descriptive statistics of the barriers toward participation depending on gender, family income, living environment and educational level are presented in Table 4. One-way MANOVA analyses revealed differences in perceived barriers between men and women (multivariate *F* = 3.232, *p* = 0.002) and between the different income groups (multivariate *F* = 2.122, *p* = 0.041). Regarding gender, univariate analyses showed that lack of interest was rather perceived as a barrier in men than in women (*F* = 9.029, *p* = 0.003), while perceiving financial barriers was more prevalent in women than in men (*F* = 4.584, *p* = 0.033). Regarding monthly family income, univariate differences between income groups were found for financial barriers (*F* = 5.218, *p* = 0.006), familiarity with the event (*F* = 4.398, *p* = 0.013) and similarities with other events (*F* = 5.596, *p* = 0.004).

**Table 4:** Perceived barriers toward participation in the Earth Hour event: descriptive statistics and differences between socio-demographic subgroups (gender, family income, educational level, living environment).

Barriers toward participation Mean (SD)^1^	Total sample (*n* = 372)	Gender		Monthly family income		
		Men *n* = 115	Women *n* = 257	Low *n* = 191	Moderate *n* = 134	High *n* = 47
Lack of time	3.56(0.92)	3.62(0.89)	3.54(0.93)	3.57(0.90)	3.49(0.90)	3.72(1.05)
Lack of interest	4.03(0.87)	**3.83(0.89)**	**4.12(0.85)**	3.99(0.89)	4.01(0.80)	4.21(0.94)
Lack of company/encouragement	3.31(1.00)	3.30(1.04)	3.32(0.98)	3.35(1.02)	3.19(0.92)	3.55(1.07)
Disappointment or doubts in effectiveness	3.62(0.96)	3.62(0.98)	3.61(0.96)	3.62(1.04)	3.52(0.82)	3.85(0.99)
Financial barriers	3.29(1.10)	**3.47(1.07)**	**3.21(1.11)**	**3.42(1.07)**^X^	**3.04(1.08)** ^X^	**3.45(1.13)**
Familiarity with the event	3.44(0.97)	3.51(1.02)	3.41(0.94)	**3.47(0.97)**	**3.29(0.90)** ^X^	**3.77(1.04)** ^X^
Similarities with other events^2^	2.71(1.00)	2.58(1.05)	2.77(0.97)	**2.64(1.00)** ^X^	**2.92(0.97)** ^X,Y^	**2.40(1.00)** ^Y^

Barriers toward participation Mean (SD)^1^	Educational level		Living environment		
	Undergraduate *n* = 225	Junior college *n* = 147	Urban *n* = 86	Suburban *n* = 99	Rural *n* = 187
Lack of time	3.55(0.94)	3.59(0.89)	3.59(1.05)	3.44(0.83)	3.61(0.90)
Lack of interest	4.01(0.91)	4.05(0.81)	3.91(0.98)	4.01(0.90)	4.09(0.79)
Lack of company/encouragement	**3.22(1.00)**	**3.46(0.98)**	3.24(1.08)	3.26(0.97)	3.37(0.98)
Disappointment or doubts in effectiveness	3.63(0.98)	3.60(0.95)	3.56(1.02)	3.59(0.92)	3.66(0.96)
Financial barriers	3.22(1.11)	3.39(1.07)	3.22(1.14)	3.20(0.96)	3.36(1.15)
Familiarity with the event	3.37(0.99)	3.54(0.93)	**3.43(0.99)**	**3.24(0.84)**	**3.55(1.00)**
Similarities with other events^2^	2.78(1.05)	2.61(0.92)	**2.76(1.04)**	**2.91(0.94)**	**2.59(1.00)**

SD = standard deviation. ^1^All barriers were scored on a five-point Likert scale from impossible to very likely. ^2^Higher index stands for less likelihood to be negatively affected by barriers, including similarities with other events. Bold results represent significant differences between groups (gender, monthly family income, educational level or living environment); *post hoc* tests for income group: same superscript characters (X,Y) = significant difference between groups.

*Post-hoc* analyses showed that similarities with other events was rather perceived as a barrier in students from extreme income groups (low: *p* = 0.039, high: *p* = 0.007) than students with moderate monthly family income. Low familiarity with the event was more prevalent in students from moderate-income families than in those from high-income families (*p* = 0.01). Furthermore, low-income group was less likely to be affected by financial barriers regarding this event than moderate income group (*p* = 0.007). For educational level and living environment, the multivariate model was non-significant (*F* = 1.168, *p* = 0.321 and *F* = 1.032, *p* = 0.418 respectively). However, univariate analyses showed that lack of company was more prevalent in undergraduates than in junior college students (*F* = 4.885, *p* = 0.028); and univariate differences concerning living environment were found for familiarity with the event (*F* = 3.264, *p* = 0.039) and similarities with other events (*F* = 3.285, *p* = 0.039).

### 4.4 Ranking of perceived barriers toward participation in the total sample and in students with characteristics associated with extreme odds of participating in Earth Hour event

Table 5 shows a ranking based on the importance (i.e., average item scores) of each potential barrier preventing participation in the different subgroups. In the total sample (*n* = 372), the top three of perceived barriers toward participation consisted to similarities with other events, financial barriers, and lack of company. This stands true for students who participated in Earth Hour for at least one time during the past 5 years and those who never participated as well. In non-participants, lack of company or encouragement was relatively prior to financial barriers compared with the participants. Furthermore, just like in the total sample, lack of time, doubts in effectiveness and lack of interest completed the last three barriers, except in participants who believe that low familiarity with the event was less important than lack of time.

**Table 5:** Ranking of perceived barriers toward participation in the total sample and in students with characteristics associated with extreme odds of participating in Earth Hour event.

Barriers toward participation^1^	Total sample (*n* = 372) Rank (Mean [SD])	Non-participants (*n* = 174) Rank (Mean [SD])	Participants (*n* = 198) Rank (Mean [SD])	Low social support from family and friends^A^ (*n* = 132) Rank (Mean [SD])
Similarities with other events^2^	1(2.71[1.00])	1(2.93[1.03])	1(2.53[0.94])	4(3.25[0.84])
Financial barriers	2(3.29[1.10])	3(3.22[1.09])	2(3.34[1.10])	3(3.00[1.04])
Lack of company/encouragement	3(3.31[1.00])	2(3.06[1.04])	3(3.54[0.90])	1(2.72[0.85])
Familiarity with the event	4(3.44[0.97])	4(3.24[0.99])	5(3.62[0.91])	2(2.95[0.89])
Lack of time	5(3.56[0.92])	5(3.53[0.89])	4(3.59[0.95])	5(3.27[0.90])
Disappointment or doubts in effectiveness	6(3.62[0.96])	6(3.60[0.98])	6(3.63[0.95])	6(3.37[1.00])
Lack of interest	7(4.03[0.87])	7(3.95[0.92])	7(4.10[0.81])	7(3.81[1.00])

SD = standard deviation. ^1^All barriers were scored on a five-point Likert scale from impossible to very likely. ^2^Higher index stands for less likelihood to be negatively affected by barriers, including similarities with other events; A median split was used to define groups scoring “low/high” on the respective psychosocial variables.

Based on the results of the binary logistic regression analysis (study aim 1), participants scoring “low” on social support associated with participation were selected as our focus on low participation analysis. This was done using a median split (i.e., selection of participants scoring lower than the median score) for all psycho-social factors. Compared with the total sample, non-participant group and participant group, students gaining low support from family and friends were more likely to be baffled by inadequate familiarity with the event than excessive similarity with other events in the low social support group. In other words, lower support from family or friends might prevent college students from knowing this event, which enhanced the negative impact of this barrier on participation.

## 5 Discussion

Initial sociological studies on Earth Hour participation in PRC focused on cities and institutions, suggesting a high (i.e., above 60% in 1 year) participation rate (HuiCong D&B Research, 2011). More recent work based on a smaller sample, in contrast, demonstrated that individuals’ practice in this event is less active than expected (Wang, 2015). Our data establish that more occasional participants were found than regular participants during the past 5 years among Chinese college students, with a lower level of average participation frequency (i.e., 1.03: 5) compared with the previous investigation (i.e., 2.7: 8) (Wang, 2015). Notably, this finding is in line with the existing statistics which has implied a descending trend of participation in Earth Hour nationwide, however, applying a new research method that is more theoretical based (Ajzen, 1985; Van Dyck et al., 2017; Chan et al., 2020).

The findings in this study are in accordance with Van Dyck’s study (Van Dyck et al., 2017) when it comes to socio-demographic factors, as no significant differences in participation were found according to gender, monthly family income, educational level and living environment. This is a positive trend and denies the results that rural residents are less inclined to participate in Earth Hour due to its limited exposure in the countryside (Wang, 2015); the finding also supports the latest statistics regarding a rapid surge in participants from third-tier and fourth-tier cities since the gap is no longer significant (Zhuang, 2023). Besides, this also indicates a narrowing gender disparity in domestic environmental participation, contrasting with Li et al. (2022) findings that contemporary Chinese women are more actively engaged in PEB activities compared with men. It also validates the conclusion that gender differences are not significant in college students’ environmental participation activities (Vicente-Molina et al., 2018). Among non-participants, the top three barriers were similarities with other events, financial barriers, and lack of company. However, it’s doubtful whether financial barrier is a credible enough to measure students’ participation since students with different levels of monthly family income were rather similar in all barrier ratings. Despite the doubts in effect of reducing electricity consumption in Earth Hour being one of the main reasons for non-participation overseas (Vuong et al., 2020), no clear evidence supportive of this finding was found in our study since disappointment or doubts in effectiveness ranked second to last in the total sample, non-participants and students with characteristics associated with extreme odds of participation (see section 3.4).

No previous studies examined the mismatch between respondents’ belief and practice in relevant activities nationwide, particularly, among college students whose behaviors is yet likely to be influenced by higher education. Distinguished from Yang and Li (2021), environmental awareness which is commonly known as a booster for environmental protection activities was not found to be positively related to students’ participation. One possible explanation is that the environmental protecting education in China might not be effective enough to make a difference in students’ behaviors for schools have put excessive emphasis on plain theoretical education without giving specific instructions or useful advice on how to put it into practice.

A highlight of findings regarding the psychosocial correlates of participation would be the positive relation between participation and social support with a considerable level of significance found (*p* < 0.01), verifying the results from Yang and Li (2021). To be more specific, participation rate increased by 76.32% with each additional unit of social support from family and friends. Sadly, the participation rate in Earth Hour remains low nationwide (see section 3.1), which makes it harder for participants to maintain their passion for this event. In other words, the herd effect has led to a vicious cycle that worsens this issue. To some extent, this is partly due to the immature build of local NGO (i.e., Non-government Organization) concerning environmental protection, in other words, bottom-up attempts are to be taken seriously besides other top-down approach mentioned in previous studies that investigated the institutional framework to allow public participation (Li et al., 2012).

When it comes to potential impact on barriers toward participation by socio-demographic variables, the experienced barriers were relatively similar across subgroups concerning gender, income, educational level and living environment, with some exceptions. For women, having a poor economic condition was more important while men rather considered this event dull, which agreed with the domestic finding that females exhibited a greater propensity for environmental concerns (Li et al., 2022). Furthermore, financial barriers and being unfamiliar to the event were mainly present in individuals with moderate income while boredom caused by similarities to other events had a greater impact on extreme income subgroups. This finding supported existing PEB studies (Margetts and Kashima, 2017; Chatelain et al., 2018) by explaining how similarities with other events applied to specific populations as one of the perceived barriers in Earth Hour. College students better educated were more likely to be affected by people around them. Those who lived in rural area shared a higher level of exposure to this event and named less similar events, which indicated that its publicity could be greatly improved in countryside despite less attention it had raised in cities during the past decade (Wang, 2015).

Finally, an overview of the event-specific barriers preventing participation was given in both the total sample and students with characteristics associated with lower odds for participation. In the overall sample, the main three barriers were similarities to other events, financial barriers, and lack of company. Except for students with lower social support from family and friends, similarities to other events completed the top. When looking specifically at the main barriers in students who are less likely to participate in Earth Hour, lack of company and low familiarity with the event were more prevalent, and results were rather similar as for barriers ranked the bottom three.

This study gives insights to researchers working on participation analysis of large-scale event in broader socio-ecological context. It highlights the dual nature of interplay between individual and social environment in group activities held with a large population. Being indifferent and less motivated to participate in such events would cause a herd effect among participants, thus reducing the participation rate in overall. On the other hand, boosting social support would be the key to end the negative feedback and create a virtuous circle instead.

Although this study focused on PRC college students, it has wider implications for many school educators, practitioners and organizations involved in pro-environmental career. The conflict between students’ belief and practice in Earth Hour indicates a lack of efficiency in local pro-environmental education, leading curriculum planners’ reflection on how to motivate students to engage in PEB via innovative lessons. An effective approach to tackle this problem was mentioned by Jin et al. (2023), where a whole set of adapted curricula covering green perspective was highly recommended rather than separate thematic lectures. For pro-environmental institutes, reinforcing the NGO construction and encouraging the current participants to join in groups is the key to reducing the non-participants since a strong positive correlation was found between social support and participation. Given the high priority of similarities with other events, features unique to Earth Hour are expected to be emphasized to raise public awareness; for local male participants, extra new forms should be advocated in relevant pro-environmental activities as an effort to cater to their interest.

From an international perspective, this paper shed light on the essence of pro-environmental collective action similar to Earth Hour, which is to convert unconcerned members in the general public into active members in the environmental endeavors. The research not only elucidated local dynamics but also contributed to a global dialogue on fostering sustainable behaviors, crucial in mitigating climate change’s adverse effects and safeguarding our planet for future generations.

## 6 Conclusion

In conclusion, this study confirmed the low participation among Chinese college students in Earth Hour event. Despite that no significant mismatch was shown between students’ belief and practice on environmental protection, the irrelevance warned that the effect of publicity and education involved were far from ideal. It also showed that low levels of social support were associated with a lower likelihood of participating in the earth hour. Furthermore, similarity with other events, financial barriers and lack of company were the three main barriers preventing Chinese college students from participation. Finally, with a few exceptions, perceived barriers were relatively similar across socio-demographic subgroups.

This study has limitations, naturally. Perhaps the most general limitation arose during the analysis of the questionnaires, that respondents might overstate their engagement. Similar cases were discussed in a study on Earth Hour participation among Sydney residents (Solomon, 2008), which suggested a 36% overstated participation rate potentially triggered by moral cost and pressure from scrutiny according to the model elaborated by Levitt and List (2007). A minor problem would be the error caused by the misconduct in sample collection. Most regular participants (29 out of 401) were excluded from the total sample, which undermined the accuracy of analysis. In addition, the failure to compare respondents’ engagement in Earth Hour with that in other types of environmental protection activities is a limitation, otherwise, problems unique to Earth Hour might have been better noticed.

For future studies, an ambiguous question is that whether the herd effect found in our research was a matter unique to the event or to the local population size. Thus, the relation between social support and participation rate is recommended to be analyzed separately under the following two situations: (1) same activity on sparsely populated area; (2) less population-based activities with a similar sample included in this study. In addition, measuring participation in a quantitative way with other methods (if necessary), such as the lasting time for switching lights off, continuity of annual participation or the alternative ways participants take part in this event would be an interesting extension to this study.

## Data availability statement

The original contributions presented in the study are included in the article/Supplementary material, further inquiries can be directed to the corresponding author.

## Ethics statement

The studies involving humans were approved by the Shanghai Normal University. The studies were conducted in accordance with the local legislation and institutional requirements. The participants provided their written informed consent to participate in this study. Written informed consent was obtained from the individual(s) for the publication of any potentially identifiable images or data included in this article.

## Author contributions

KY: Visualization, Software, Resources, Methodology, Investigation, Formal analysis, Data curation, Conceptualization, Writing – original draft, Writing – review & editing. YW: Writing – review & editing. HX: Writing – review & editing. ML: Supervision, Validation, Resources, Methodology, Project administration, Conceptualization, Writing – original draft, Writing – review & editing.
