# Supplementary material for: Socio-ecological model as a framework to understand the low participation of Earth Hour among Chinese college students: conflict between belief and practice
Source: Front Psychol. 2024 May 6;15:1288711. doi: 10.3389/fpsyg.2024.1288711 (PMC11104500; doi:10.3389/fpsyg.2024.1288711)
Supplement: Supplementary file 1 [file Data_Sheet_1.doc]

**Appendix: Questionnaire**

[Participation]

1.How often did you participate in the Earth Hour in the past five years?

A. Never

B. 1~2 times

C. 3~5 times

[Socio-demographic factors]

2.Your gender:

A. Male

B. Female

3.Your monthly family income:

A. Below￥5000

B. Between ￥5000 and ￥10000

C. Above￥10000

4.Which place had you lived for the longest time before you lived on campus in Shanghai?

A. Urban area (city center despite the level of city)

B. Suburban area (satellite city included)

C. Rural area

5.Which category does your university belong to?

A. “985” Universities

B. “211” Universities

C. General first-class Universities

D. Universities below first-class

E. Colleges

[Psychosocial factors]

6~7 SA for strongly agree；A for agree；U for undecided(or if you do not understand the statement)；D for disagree；SD for strongly disagree.

*----- 6. It’s of great importance to protect general resource.

*----- 7. The existing domestic laws on the implementation and promotion of large-scale environmental protection activities are perfect.

8 N for never; SE for seldom; SO for sometimes; O for often; A for always.

*----- 8. How often did your family members/friends participate/encourage you to participate/participate with you in the Earth Hour?

9. When should you turn off your lights on the last Saturday in March?

A. Between 9:30 pm and 10:30 pm.

B. Between 11.pm and midnight.

C. Between 7:30 pm and 8:30 pm.

D. Between 8:30 pm and 9:30 pm.

E. Between 10:30 pm and 11:30 pm.

10.Where did the first Earth Hour take place in 2007?

A. Portland, Oregon

B. Oslo, Norway

C. Copenhagen, Denmark

D. Toronto

E. Sydney, Australia

11.Why does Earth Hour take place at the end of March?

A. To coincide with Earth Day

B. To celebrate the arrival of spring

C. The days get longer, making one hour of dark more bearable

D. It allows for near-coincidental sunset times in both hemispheres

E. Summer and winter Earth Hour were not as successful

12.Which group organizes Earth Hour every year?

A. Earth watch

B. European Environment Agency

C. Earth First

D. World Nature Organization

E. Worldwide Fund for Nature

[Barriers toward participation]

13~19 I for impossible; NL for not likely; U for undecided; P for possible; VL for very likely.

*-----13. I’m willing to concern about public activities related to environmental protection.

*-----14. I would rather spend time on the long-term goals with more return than on my current needs.

*-----15. People around me actively participate in Earth Hour environmental protection activities.

*-----16. [Alternative] A. Earth hour has no potential electric power hazard or negative impact on the environment.

B. There is no conflict between the publicity of environmental awareness and the implementation efficiency in environmental protection activities.

*-----17. Poverty does not make me burn the midnight oil.

*-----18. Content related to Earth Hour are often involved in the media, books, and teaching by example that I meet.

*-----19. I can name at least more than three activities that are similar to Earth Hour.
